# The *microRNA-23b/27b/24-1* cluster is a disease progression marker and tumor suppressor in prostate cancer

**DOI:** 10.18632/oncotarget.2294

**Published:** 2014-07-31

**Authors:** Yusuke Goto, Satoko Kojima, Rika Nishikawa, Hideki Enokida, Takeshi Chiyomaru, Takashi Kinoshita, Masayuki Nakagawa, Yukio Naya, Tomohiko Ichikawa, Naohiko Seki

**Affiliations:** ^1^ Department of Functional Genomics, Chiba University Graduate School of Medicine, Chiba, Japan; ^2^ Department of Urology, Chiba University Graduate School of Medicine, Chiba, Japan; ^3^ Department of Urology, Teikyo University Chiba Medical Center, Chiba, Japan; ^4^ Department of Urology, Graduate School of Medical and Dental Sciences, Kagoshima University, Kagoshima, Japan

**Keywords:** microRNA, miR-23b, miR-27b, miR-24-1, castration-resistant prostate cancer

## Abstract

Our recent study of microRNA (miRNA) expression signatures in prostate cancer (PCa) has revealed that all members of the *miR-23b/27b/24-1* cluster are significantly downregulated in PCa tissues. The aim of this study was to investigate the effectiveness of these clustered miRNAs as a disease progression marker and to determine the functional significance of these clustered miRNAs in PCa. Expression of the *miR-23b/27b/24-1* cluster was significantly reduced in PCa tissues. Kaplan-Meier survival curves showed that low expression of *miR-27b* predicted a short duration of progression to castration-resistant PCa. Gain-of-function studies using mature *miR-23b*, *miR-27b*, and *miR-24-1* significantly inhibited cell proliferation, migration and invasion in PCa cells (PC3 and DU145). To identify the molecular targets of these miRNAs, we carried out gene expression and *in silico* database analyses. *GOLM1* was directly regulated by *miR-27b* in PCa cells. Elucidation of the molecular targets and pathways regulated by the tumor-suppressive *microRNAs* should shed light on the oncogenic and metastatic processes in PCa.

## INTRODUCTION

Prostate cancer (PCa) is the most frequently diagnosed cancer and the second leading cause of cancer-related deaths among men in developed countries [1]. Androgen signaling through the androgen receptor (AR) is an important oncogenic pathway for PCa progression. Most patients are initially responsive to androgen deprivation therapy (ADT), but their cancers eventually become resistant to ADT and progress to castration-resistant prostate cancer (CRPC). Although prostate-specific antigen (PSA) has been used for monitoring CRPC, outcomes in PCa patients are diverse, even within the same risk group, because of the heterogeneity of PCa cells [2-4]. Thus, identification of effective biomarkers for detection of CRPC is needed. With currently available therapies, CRPC is difficult to treat, and most clinical trials for advanced PCa have shown limited benefits, with disease progression and metastasis to bone or other sites [5, 6]. Therefore, understanding the molecular mechanisms of androgen-independent signaling and metastatic signaling pathways underlying PCa using current genomic approaches would help to improve therapies for and prevention of the disease.

A growing body of evidence indicates that microRNAs (miRNAs) also contribute to the initiation, development, and metastasis of various types of cancers [7]. Many human cancers show aberrant expression of miRNAs that can function as either tumor suppressors or oncogenes. Therefore, identification of aberrantly expressed miRNAs is the first step toward elucidating miRNA-mediated oncogenic pathways in human cancers. Based on this point, our group previously established miRNA expression signatures and identified novel oncogenic pathways regulated by tumor-suppressive microRNAs in several types of cancers, including PCa [8-11].

In a recent study from our laboratory, we found that the *miR-23b/27b/24-1* cluster was significantly downregulated in PCa [11]. Some miRNAs are located in close proximity on the human genome; these are termed clustered miRNAs. In the human genome, 247 human miRNAs have been found to be clustered at 64 sites at inter-miRNA distances less than 5000 bp [12, 13]. We previously reported that *miR-1-1/133a-2* and *miR-1-2/133a-1* formed clusters in different chromosomal loci in the human genome (20q13.33 and 18q11.2, respectively) and that these clusters function as tumor suppressors, targeting several oncogenic genes in human cancers, including PCa [13-15]. More recently, we showed that the *miR-143/145* cluster, located at the 5q32 locus, acts as a tumor-suppressive miRNA cluster in renal cell carcinoma and PCa [16, 17].

In this study, we hypothesized that the *miR-23b/27b/24-1* cluster functioned as a tumor suppressor by targeting several oncogenic genes involved in specific cancer-related pathways in PCa. Elucidation of the molecular targets regulated by the tumor-suppressive *miR-23b/27b/24-1* cluster will provide new insights into the potential molecular mechanisms of PCa oncogenesis and metastasis and will facilitate the development of novel diagnostic and therapeutic strategies for the treatment of PCa.

## RESULTS

### Expression of the *miR-23b/27b/24-1* cluster in PCa specimens and cell lines

The public miRNA database (miRbase: release 21) shows that *miR-23b/27b/24-1* cluster is located in close proximity on the human chromosome 9q22.32 region within 900 base pairs. The mature sequence of *miR-23b* is 5'-AUCACAUUGCCAGGGAUUACC-3', *miR-27b* is 5'-UUCACAGUGGCUAAGUUCUGC-3' and *miR-24-1* is 5'-UGGCUCAGUUCAGCAGGAACAG-3'. This clustered miRNAs are involved in *C9orf3* in their host gene.

We evaluated the expression levels of the clustered miRNAs (*miR-23b*, *miR-27b* and *miR-24-1*) in noncancerous tissues (n = 41) and PCa tissues (n = 49). In patients from whom normal prostate tissues were collected, the median PSA level was 7.3 ng/mL (range: 4.3–35.5 ng/mL). In contrast, in patients from whom PCa tissues were collected, PSA levels were quite high, with a median of 244 ng/mL (range: 3.45–3750 ng/mL). Thirty-nine PCa patients had progressive disease classified as N1 or M1 according to TNM classification (Table 1).

**Table 1:** Patients' characteristics

Characteristic	PCa (n = 49)	non-PCa (n = 41)
Age (years)		
Median (range)	73 (58-88)	63 (52-85)
PSA (ng/ml)		
Median (range)	244 (3.45-3750)	7.3 (4.3-35.5)
cT stage		
T2c	1	
T3a	12	
T3b	18	
T4	18	
cN stage		
N0	15	
N1	34	
cM stage		
M0	19	
M1	30	
Gleason Score		
3+4	1	
4+3	3	
4+4	30	
4+5	14	
5+4	0	
5+5	1	

**Figure 1:**
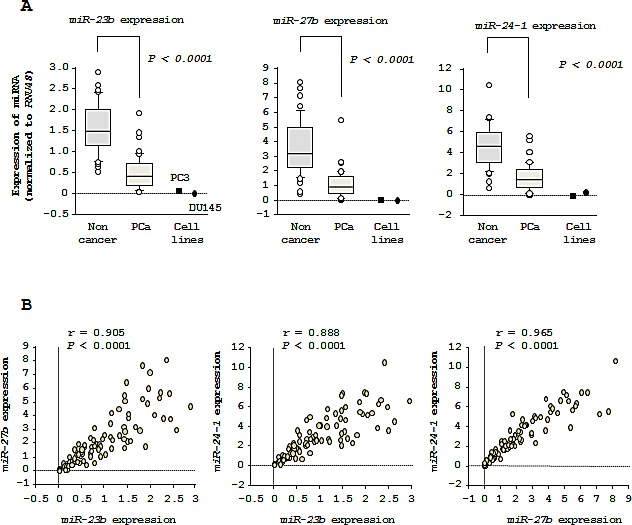
Expression levels of clustered *miR-23b/27b/24-1* in PCa specimens (A) Expression levels of *miR-23b*, *miR-27b*, and *miR-24-1* in PCa clinical specimens. *RNU48* was used for normalization. (B) Correlations among the relative expression levels of *miR-23b/miR-27b*, *miR-23b/miR-24-1*, and *miR-27b/miR-24-1*.

The expression levels of *miR-23b*, *miR-27b*, and *miR-24-1* were significantly downregulated (*P* < 0.0001) in tumor tissues as compared to corresponding noncancerous tissues (Figure 1A). Spearman's rank test showed positive correlations between the expression of *miR-23b* and *miR-27b* (*R* = 0.905 and *P* < 0.0001) and the expression of *miR-23b* and *miR-24-1* (*R* = 0.888 and *P* < 0.0001). Similarly, the expression of *miR-24-1* was positively correlated with that of *miR-27b* (*R* = 0.965 and *P* < 0.0001; Figure 1B).

### Correlations between *miR-23b/27b/24-1* expression and clinicopathological features in PCa specimens

**Figure 2:**
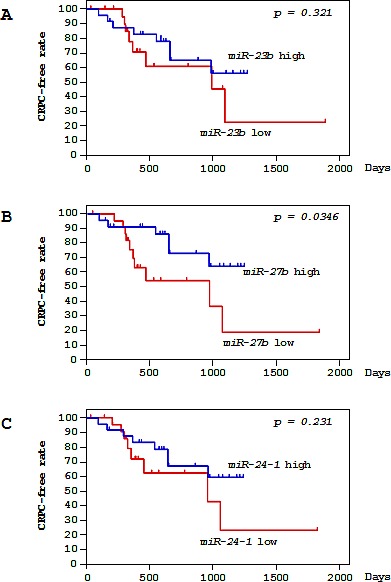
Correlations of *miR-23b/27b/24-1* expression and clinicopathological features in PCa specimens Kaplan-Meier survival curves for CRPC progression-free survival based on (A) *miR-23b*, (B) *miR-27b*, and (C) *miR-24-1* expression in PCa patients. *P*-values were calculated using the log-rank test.

Among 49 PCa patients, 47 patients underwent ADT with luteinizing hormone-releasing hormone (LHRH) agonists and anti-androgens (Supplemental Table 1). A total of 16 ADT-treated patients progressed to CRPC over a median follow-up of 15.6 months. For patients with high versus low *miR-23b/27b/24-1* expression, the risk of progression to CRPC was evaluated using the Kaplan-Meier method and log rank test for significant separation of survival curves. Low expression of *miR-27b* was found to be associated with shorter progression-free interval (*P* = 0.0346; Figure 2B). However, neither *miR-23b* nor *miR-24-1* predicted the time to CRPC in these PCa patients (*P* = 0.321 and *P* = 0.231, respectively; Figure 2A and 2C). The multivariate Cox proportional hazards model was used to assess independent predictors of time to progression to CRPC, including Gleason score, cT stage, cN stage, cM stage, PSA, age, and *miR-27b* expression. *miR-27b* expression was the only independent prognostic factor of patient outcomes for PCa patients treated with ADT (hazard ratio [HR]: 0.255, 95% confidence interval [CI]: 0.069-0.944, *P* = 0.0407; Table 2).

**Table 2:** Cox proportional analysis for the prediction of CRPC progression-free survival

Covariant	HR	95% CI	*P-value*
*miR-27b*	0.255	0.069-0.944	0.0407
Gleason score	2.003	0.747-5.374	0.1674
cT stage	2.111	0.490-9.098	0.3162
cN stage	2.28	0.372-13.975	0.3729
cM stage	1.021	0.203-5.124	0.9799
PSA	1.001	1.000-1.001	0.0719
Age	1.045	0.949-1.150	0.3674

### Effects of restoring *miR-23b/27b/24-1* expression on cell proliferation, migration, and invasion in PC3 and DU145 PCa cells

To investigate the functional effects of the *miR-23b/27b/24-1* cluster, we performed gain-of-function studies using miRNA transfection in 2 PCa cell lines (PC3 and DU145).

**Figure 3:**
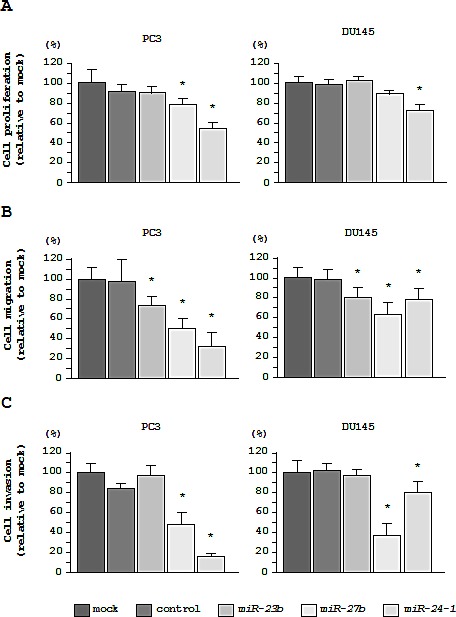
Effects of *miR-23b/27b/24-1* transfection on cell proliferation, migration, and invasion in PC3 and DU145 cells (A) Cell proliferation 72 h after transfection with *miR-23b/27b/24-1* was determined using XTT assays. (B) Cell migration activity 48 h after transfection with *miR-23b/27b/24-1* was determined using migration assays. (C) Cell invasion activity 48 h after transfection with *miR-23b/27b/24-1* was determined using Matrigel invasion assays. *P < 0.005.

As observed using XTT assays, cell proliferation was significantly inhibited in *miR-27b* and *miR-24-1* transfectants as compared with mock- or miR-control-transfected PC3 cells. However, the *miR-23b* transfectant did not exhibit reduced cell proliferation in PC3 cells. In contrast, inhibition of cell proliferation was only observed in the *miR-24-1* transfectant in DU145 cells (Figure 3A).

In cell migration assays, transfection with each of the 3 miRNAs significantly inhibited cancer cell migration in all cell lines (Figure 3B).

In cell invasion assays, transfection with *miR-27b* and *miR-24-1* inhibited cell invasion in PC3 and DU145 cells. However, transfection with *miR-23b* did not inhibit cell invasion in either PC3 or DU145 cells (Figure 3C).

In this study, we investigated the synergistic effects of *miR-23b/27b/24-1* cluster in PCa cells. We performed XTT and migration assays using transfection of all possible combinations of these clustered miRNAs. However, we did not find synergistic effects in these assays (Supplemental Figure 1).

### Identification of target genes regulated by the *miR-23b/27b/24-1* cluster in PCa

To gain further insights into the molecular pathways and targets regulated by the tumor-suppressive *miR-23b/27b/24-1* cluster in PCa, we first identified putative target genes of *miR-23b, miR-27b*, and *miR-24-1* by searching the TargetScan database. This analysis identified 4206, 4075, and 4321 putative target genes for *miR-23b*, *miR-27b*, and *miR-24-1*, respectively.

Next, we investigated the expression statuses of these putative targets in PCa clinical specimens and examined gene expression profiles in the GEO database (accession numbers GSE29079) to evaluate upregulated genes in PCa specimens. Among the 4206 putative target genes of *miR-23b*, 147 genes were significantly upregulated in PCa specimens compared to noncancerous prostate tissues. In a similar analysis, among 4075 and 4321 putative targets of *miR-27b* and *miR-24-1*, 157 and 139 genes were upregulated in PCa tissues, respectively.

**Figure 4:**
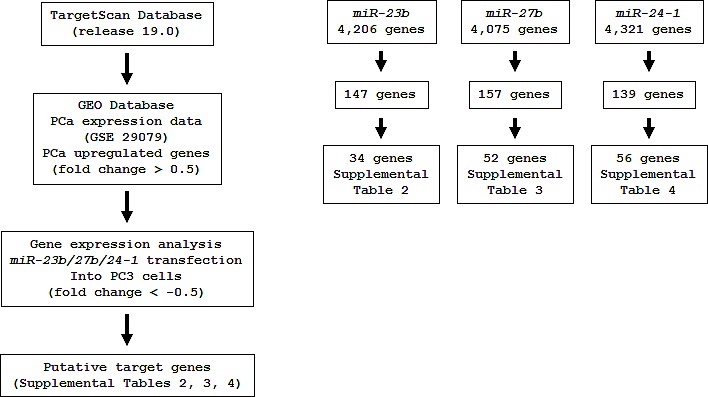
Flow chart of the strategy for analysis of *miR-23b/27b/24-1* cluster target genes A total of 4206, 4075 and 4321 genes were selected as putative *miR-23b, 27b and 24-1* target genes, respectively by TargetScan database analysis. We then analyzed the expression levels of these candidate genes by using available data sets of GEO (GSE 29079). The analyses showed that 147, 157 and 139 genes were significantly upregulated in PCa specimens compared with normal specimens. Furthermore, expression analysis data of *miR-23b*, *miR-27b* and *miR-24-1* transfectants of PC3 cells were merged. Finally, 34, 52 and 56 genes were putative candidate genes regulated by *miR-23b*, *miR-27b* and *miR-24-1*, respectively.

Furthermore, we performed genome-wide gene expression analysis using PC3 cells and selected genes that were downregulated following transfection with *miR-23b/27b/24-1* as compared with the miR-control. Entries from the gene expression data were approved by GEO, and were assigned GEO accession number GSE47657. When we integrated all of these analysis results, a total of 34, 52, and 56 genes were identified as targets of *miR-23b*, *miR-27b*, and *miR-24-1*, respectively (Supplemental Tables 2-4). Our strategy for selection of *miR-23b/27b/24-1* cluster-targeted genes is shown in Figure 4.

### *GOLM1* was a direct target of *miR-27b* in PCa cells

**Figure 5:**
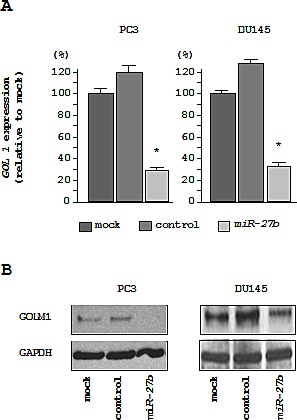
Downregulation of *GOLM1* expression by *miR-27b* in PC3 and DU145 cells (A) *GOLM1* mRNA expression 72 h after transfection with *miR-27b*. GUSB was used as an internal control. *P < 0.005. (B) GOLM1 protein expression 72 h after transfection with *miR-27b*. GAPDH was used as a loading control.

We performed real-time RT-qPCR and western blotting in PC3 and DU145 cells to investigate whether restoration of *miR-27b* altered the expression of the *GOLM1* gene and GOLM1 protein. The mRNA and protein expression levels of *GOLM1*/GOLM1 were significantly repressed in *miR-27b* transfectants as compared with mock- or miR-control-transfected cells (Figures 5A and 5B).

**Figure 6:**
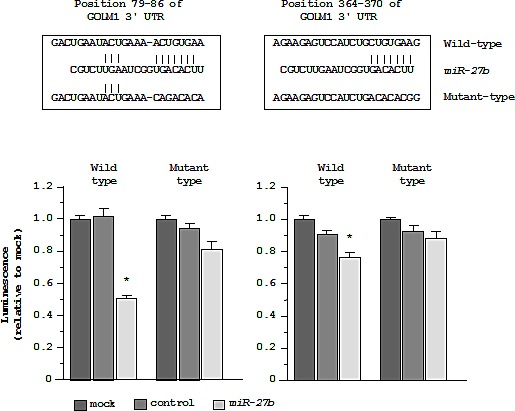
Regulation of *GOLM1* expression by *miR-27b* *miR-27b* binding sites in *GOLM1* mRNA. Luciferase reporter assay using the two vectors encoding putative *miR-27b* target sites of the *GOLM1* 3'-UTR (positions 79-86 and 364-370) for both wild-type and mutant constructs. *P < 0.005.

Therefore, we next performed luciferase reporter assays in PC3 cells to determine whether *GOLM1* mRNA had target sites for *miR-27b*. The TargetScan database predicted that 2 putative *miR-27b* binding sites existed in the 3'UTR of *GOLM1* (positions 79-86 and 364-370). We used vectors encoding a partial wild-type sequence of the 3'UTR of *GOLM1* mRNA, including the predicted *miR-27b* target site or a vector lacking the *miR-27b* target site. We found that the luminescence intensity was significantly reduced by cotransfection with *miR-27b* and the vector carrying the wild-type 3'UTR of *GOLM1*. On the other hand, the luminescence intensity was not decreased when the seed sequence of the target site was deleted from the vectors (Figure 6).

### *GOLM1* expression in PCa specimens

**Figure 7:**
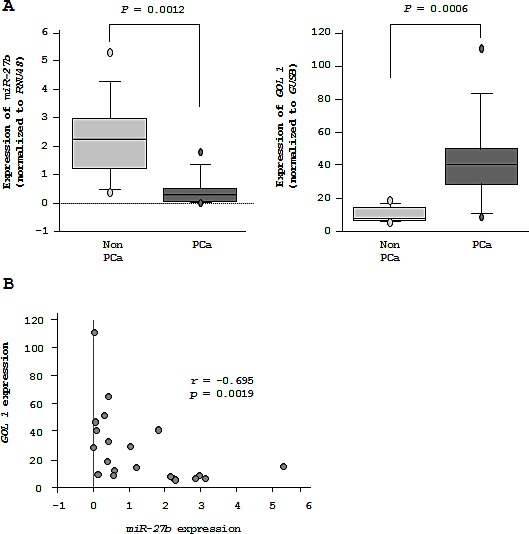
Expression of *GOLM1* and *miR-27b* in PCa specimens (A) *miR-27b* and *GOLM1* mRNA expression in 10 non-PCa specimens and 11 clinical PCa specimens. (B) Inverse correlation between *GOLM1* mRNA and *miR-27b* expression.

Among 90 PCa and non-PCa samples, we selected RNA samples that could be used for reverse transcription analysis of *GOLM1* mRNA expression. Finally, 11 PCa samples and 10 non-PCa samples were subjected to *GOLM1* mRNA expression analysis in this study. RT-qPCR showed that *miR-27b* expression was significantly lower in PCa samples compared with non-PCa samples (*P* = 0.0012, Figure 7A). Moreover, the expression of *GOLM1* was significantly higher in PCa tissues compared with normal tissues (*P* = 0.0006, Figure 7A). Spearman's rank test showed that the lower expression of *GOLM1* was correlated with higher *miR-27b* expression (*r* = -0.695 and *P* = 0.0019, Figure 7B).

## DISCUSSION

Aberrantly expressed miRNAs disrupt the tightly regulated RNA networks in cancer cells, triggering cancer development and metastasis. Therefore, identification of the differentially expressed miRNAs in cancer cells is the first step to elucidating novel miRNA-mediated pathways in cancer. Expression signatures of various types of cancer tissues are important sources for the study of miRNAs in cancer. Based on the observed miRNA signatures, our group has identified tumor-suppressive miRNAs in PCa [11, 15, 17]. In this study, we focused on the *miR-23b/27b/24-1* cluster because all of these miRNAs have been reported as downregulated miRNAs in our PCa signatures [11]. This is the first report that aimed to investigate the functional significance of all members of the *miR-23b/27b/24-1* cluster in PCa. We confirmed the downregulation of the *miR-23b/27b/24-1* cluster in PCa tissues with sets of independent clinical specimens. Elucidation of several miRNA signatures in PCa has demonstrated that some members of this miRNA cluster are expressed at low levels in cancer tissues [18-20]. From our analysis, we found that the miRNAs within the *miR-23b/27b/24-1* cluster were regulated by the same transcriptional control mechanism in the human genome. A recent report showed that the transcription factor AP-1 directly binds to the promoter region of *miR-23b* and reduces its expression in MDA-MB-231 cells [21]. Interestingly, the expression of AP-1 (c-Jun and c-Fos) is associated with aggressive and androgen-independent prostate cancer [22, 23]. While the mechanism silencing the expression of the *miR-23b/27b/24-1* cluster in PCa cells is still unknown, analysis of the detailed molecular mechanisms will be necessary in future studies.

Current PCa screening methods are based on the measurement of serum PSA, and a definite diagnosis is established by ultrasound-guided prostate needle biopsies [24, 25]. PSA is the most common marker for detection of PCa and for following the course of CRPC or metastatic PCa. However, the course of PCa progression and clinical outcomes of PCa patients can differ even in patients with the same PSA value, Gleason score, and pathological stage. Thus, it is crucial to identify more sensitive biomarkers for improvement of PCa prognosis. Several groups have described the independent biochemical recurrence of prediction markers based on the expression levels of miRNAs, such as *miR-21*, *miR-145*, *miR-200a*, and *miR-30d* [26-29], using radical prostatectomy specimens. If the expression status of miRNAs derived from needle biopsies at the initial visit can predict the possibility of progression to CRPC or metastasis, physicians can make more accurate treatment decisions for PCa patients. To investigate this, we analyzed the expression levels of miRNAs within the *miR-23b/27b/24-1* cluster and their associations with the clinicopathological features of PCa patients. Surprisingly, the expression status of *miR-27b* indicated that this miRNA was a good prognostic marker for time to progression to CRPC. A large-scale cohort study will be necessary to determine whether *miR-27b* is an effective marker for CRPC-free interval.

Our present data demonstrated that restoration of the *miR-23b/27b/24-1* cluster significantly inhibited cancer cell proliferation, migration, and invasion in both androgen-dependent and –independent PCa cells, suggesting that the *miR-23b/27b/24-1* cluster functioned as a tumor suppressor in PCa. Several studies have reported that these miRNAs have tumor-suppressive roles in PCa cells, similar to the results of our present study. For example, *miR-23b* directly controls the proto-oncogenes Src kinase and Akt, and overexpression of *miR-23b* inhibits proliferation, migration, invasion, cell cycle arrest, and apoptosis [30]. Another report has shown that *miR-23b* and *miR-27b* are downregulated in metastatic and CRPC tumors and that ectopic expression of these miRNAs suppresses cell invasion and migration in CRPC cell lines [31]. In contrast, the expression of *miR-23b* and *miR-27b* is highly upregulated in human breast cancer, and knockdown of *miR-23b* and *miR-27b* substantially represses breast cancer growth [32]. Interestingly, the expression status of the *miR-23b/27b/24-1* cluster is not consistent among different types of cancers. Elucidation of the mechanisms controlling the expression of clustered miRNAs in each cancer is an important theme in this developing field. To date, few reports have described the functional significance of *miR-27b* and *miR-24-1* in PCa cells.

Identification of the targets regulated by the tumor-suppressive *miR-23b/27b/24-1* cluster is important for clarifying our understanding of PCa oncogenesis and metastasis. Based on this view, we identified target genes of the *miR-23b/27b/24-1* cluster using a combination of *in silico* analysis and gene expression analysis with *miR-23b, miR-27b*, and *miR-24-1* transfectants. Using this strategy, we succeeded in proving that the tumor-suppressive *miR-1/133a* or *miR-143/145* cluster regulates oncogenic genes in various cancers [15-17]. In the present study, we identified putative target genes regulated by miRNAs in the *miR-23b/27b/24-1* cluster. Identification of these target genes will contribute to elucidation of novel cancer networks in PCa.

Finally, we focused on the *GOLM1* gene because this gene has been identified as a putative target of tumor-suppressive *miR-27b* and because *miR-27b* is a predictor of time to CRPC. Furthermore, our recent study demonstrated that the tumor-suppressive *miR-143/145* cluster commonly targets *GOLM1* and that silencing of *GOLM1* significantly inhibits the migration and invasion of PCa cells [17]. Our present data clearly showed that *GOLM1* was directly regulated by tumor-suppressive *miR-27b* in PCa cells. The GOLM1/GP73/GOLPH2 protein is encoded by the *GOLM1* gene, located on human chromosome 9q21.33. GOLM1 has been shown to be overexpressed in human PCa tissue [33, 34], lung adenocarcinoma [35], and hepatocellular carcinoma [36]. However, while a number of studies have demonstrated that GOLM1 is expressed in cancer cells, the exact molecular mechanisms mediating GOLM1 function remain unclear. Additionally, we investigated whether GOLM1 contributed to the oncogenesis and metastasis of PCa. Therefore, elucidation of the regulatory networks of GOLM1-mediated signaling pathways will provide important information for the development of new therapeutic strategies against cancer cell metastasis. Further research is needed to reveal the oncogenic functions of GOLM1 that are regulated by the tumor-suppressive *miR-143/145* cluster or by *miR-27b* in PCa.

In conclusions, downregulation of the *miR-23b/27b/24-1* cluster was a frequent event in PCa, and these clustered miRNAs functioned as tumor suppressors. Furthermore, *miR-27b* expression was a good disease progression marker in PCa. Elucidation of the molecular targets and pathways regulated by the tumor-suppressive *miR-23b/27b/24-1* cluster should shed light on the oncogenic and metastatic processes in PCa and lead to the development of more effective strategies for future therapeutic interventions in patients with PCa.

## METHODS

### Patients and clinical prostate specimens

Clinical prostate specimens were obtained from patients admitted to Teikyo University Chiba Medical Centre Hospital from 2008 to 2013. All patients had elevated PSA levels and had undergone transrectal prostate needle biopsy. Prostatic cancerous tissues (PCa, n = 49) and noncancerous tissues (non-PCa, n = 41) were used in this study. The patients' backgrounds and clinicopathological characteristics are summarized in Supplemental Table 1. Written consent for tissue donation for research purposes was obtained from each patient before tissue collection. Investigation has been conducted in accordance with the ethical standards and according to the Declaration of Helsinki and according to national and international guidelines. The protocol was approved by the Institutional Review Board of Teikyo University.

For pathological justification of tissue composition, a pair of needle biopsy specimens was collected from the same region as from patients in this study, and one was subjected to pathological justification; no cancerous tissue was found in non-PCa specimens. CRPC was defined according to European Association of Urology guidelines [37].

### Cell culture

Human prostate cancer cells (PC3 and DU145 cells) were obtained from the American Type Culture Collection (Manassas, VA, USA) and maintained in RPMI-1640 medium supplemented with 10% fetal bovine serum in a humidified atmosphere of 5% CO_2_ and 95% air at 37°C.

### RNA isolation

Total RNA was isolated using TRIzol reagent (Invitrogen, Carlsbad, CA, USA) according to the manufacturer's protocol. The quality of RNA was confirmed using an Agilent 2100 Bioanalyzer (Agilent Technologies, Santa Clara, CA, USA) as described previously [15, 38, 39].

### Quantitative real-time reverse transcription polymerase chain reaction (RT-qPCR)

The procedure for PCR quantification was carried out as previously described [15, 38, 39]. The expression levels of *miR-23b* (Assay ID: 000400), *miR-27b* (Assay ID: 000409), and *miR-24-1* (Assay ID: 000402) were analyzed by TaqMan quantitative real-time PCR (TaqMan MicroRNA Assay; Applied Biosystems) and normalized to *RNU48* (Assay ID: 001006). TaqMan probes and primers for *GOLM1* (P/N: Hs00213061_m1), *GAPDH* (P/N: Hs02758991_g1), and *GUSB* (P/N: Hs00939627_m1) as an internal control were obtained from Applied Biosystems (Assay-On-Demand Gene Expression Products). The ΔΔCt method was applied for calculation of the relative quantities of target genes. All reactions were performed in triplicate and included negative control reactions that lacked cDNA.

### Transfections with mature miRNA and small-interfering RNA (siRNA)

The following mature miRNA species were used in this study: Ambion Pre-miR miRNA precursor for hsa-*miR-23b* (product ID: PM10711), hsa-*miR-27b* (product ID: PM10750), and hsa-*miR-24-1* (product ID: PM12902; Applied Biosystems). RNAs were incubated with OPTI-MEM (Invitrogen) and Lipofectamine RNAiMAX reagent (Invitrogen). The transfection procedures and transfection efficiencies of miRNA in PC3 and DU145 cells were reported previously [15, 38, 39].

### Cell proliferation, migration, and invasion assays

To investigate the functional significance of the *miR-23b/27b/24-1* cluster, we performed cell proliferation, migration, and invasion assays using PC3 and DU145 cells. The experimental procedures were performed as described in our previous studies [15, 38, 39].

### Genome-wide gene expression and in silico analyses for the identification of genes regulated by the *miR-23b/27b/24-1* cluster

To gain further insights into the specific genes affected by the *miR-23b/27b/24-1* cluster, we performed a combination of *in silico* and genome-wide gene expression analyses. First, genes regulated by the *miR-23b/27b/24-1* cluster were listed using the TargetScan database as described previously [15, 38, 39]. Next, to identify upregulated genes in PCa, we analyzed a publicly available gene expression data set in the Gene Expression Omnibus (GEO, accession number: GSE29079). Finally, we performed genome-wide gene expression analysis using PC3 cells transfected with *miR-23b, miR-27b*, or *miR-24-1*. A SurePrint G3 Human GE 60K Microarray (Agilent Technologies) was used for expression profiling of each miRNA transfectant in comparison with negative control miRNA transfectants. Finally, downregulated mRNAs that contained target sites for each miRNA (*miR-23b/27b/24-1*) were listed as putative target genes for these miRNAs.

### Western blotting

Cells were harvested 72 h after transfection, and lysates were prepared. Twenty micrograms of protein lysate from each sample was separated on Mini-PROTEAN TGX gels (Bio-Rad, Hercules, CA, USA) and transferred to polyvinylidene difluoride membranes. Immunoblotting was performed with rabbit anti-GOLM1 antibodies (1:250, HPA010638, Atlas Antibodies, Stockholm, Sweden), and anti-GAPDH antibodies (1:1000, ab8245, Abcam) were used as an internal loading control. Membranes were washed and incubated with anti-rabbit IgG horseradish peroxidase (HRP)-linked antibodies (7074, Cell Signaling Technology, Danvers, MA, USA). Complexes were visualized with Clarity Western ECL Substrate (Bio-Rad, Hercules, CA, USA). The experimental procedures were performed as described in our previous studies [15, 38, 39].

### Plasmid construction and dual-luciferase reporter assay

Partial wild-type sequences of the *GOLM1* 3' untranslated region (UTR) or those with 2 deleted *miR-27b* target sites (positions 79-86 and 364-370 of the *GOLM1* 3'UTR) were inserted between the XhoI–PmeI restriction sites in the 3'UTR of the *hRluc* gene in the psiCHECK-2 vector (C8021; Promega, Madison, WI, USA). The protocol for vector construction was described previously [15, 38, 39].

### Statistical analysis

The relationships between two groups and the numerical values obtained by real-time RT–qPCR were analyzed using Mann–Whitney *U-*tests. Spearman's rank test was used to evaluate the correlation between the expressions levels of *miR-23b*, *miR-27b*, and *miR-24-1*. Relationships among more than 3 variables and numerical values were analyzed using Mann–Whitney *U-*tests after Bonferroni adjustment. Survival analysis was evaluated by the Kaplan-Meier method and log-rank tests. A multivariate Cox proportional hazards model was used to establish independent factors for survival. The chi-square test was used to analyze the relationship between *miR-23b/27b/24-1* expression and clinicopathological characteristics. The Kaplan-Meier method and the log-rank test were performed using Stat Mate software (version 4.01, ATMS Co., Tokyo, Japan), and other analyses were performed using Expert StatView (version 5, SAS Institute Inc., Cary, NC, USA).

## SUPPLEMENTARY MATERIAL FIGURE AND TABLES




