# Supplementary material for: The microRNA-23b/27b/24-1 cluster is a disease progression marker and tumor suppressor in prostate cancer
Source: Oncotarget. 2014 Jul 31;5(17):7748–59. doi: 10.18632/oncotarget.2294 (PMC4202158; doi:10.18632/oncotarget.2294)
Supplement: Supplementary file 1 [file oncotarget-05-7748-s001.pdf]

## The *microRNA-23b/27b/24-1* cluster is a disease progression marker and tumor suppressor in prostate cancer

**Supplementary Material**

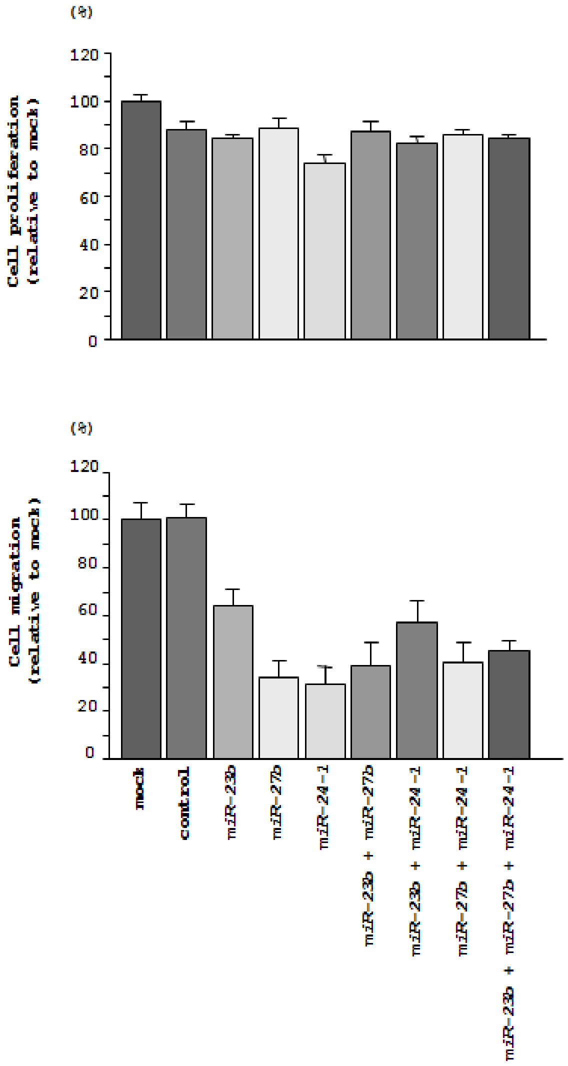


Supplemental Figure 1: Combination effects of mature *miR-23b*, *miR-27b* and *miR-24-1* transfection into PC3 cells. Pre-*miR-23b*, *miR-27b* and *miR-24-1* were used in the gain-of-function assays (XTT and migration). PC3 cells were transiently transfected with combination of these miRNAs with 10 nM for each miRNAs (*miR-23b*, *miR-27b*, *miR-24-1*, *miR-23b* + *miR-27b*, *miR-23b* + *miR-24-1*, *miR-27b* + *miR-24-1* and *miR-23b* + *miR-27b* + *miR-24-1*) using Lipofectamine RNAiMAX transfection reagent.

Supplemental Table 1: Patients' characteristics and clinicopathological features.

| No. | PCa or non-PCa | Age | PSA (ng/mL) | Gleason score | Stage | cT | cN | cM | Treatment |
|---|---|---|---|---|---|---|---|---|---|
| 1 | PCa | 67 | 244 | 4+4 | IV | 4 | 1 | 1 | ADT |
| 2 | PCa | 70 | 395 | 4+4 | IV | 3a | 1 | 1 | ADT |
| 3 | PCa | 83 | 49.9 | 4+5 | III | 3a | 0 | 0 | ADT |
| 4 | PCa | 68 | 212 | 4+4 | IV | 3b | 1 | 0 | ADT |
| 5 | PCa | 80 | 589 | 4+4 | IV | 3b | 1 | 0 | ADT |
| 6 | PCa | 72 | 2530 | 4+5 | IV | 3a | 1 | 1 | ADT |
| 7 | PCa | 76 | 12.5 | 4+5 | IV | 3b | 1 | 1 | ADT |
| 8 | PCa | 67 | 153 | 4+4 | IV | 4 | 1 | 1 | ADT |
| 9 | PCa | 82 | 808.8 | 4+4 | IV | 4 | 1 | 1 | ADT |
| 10 | PCa | 88 | 50.5 | 4+4 | III | 3a | 0 | 0 | ADT |
| 11 | PCa | 69 | 3.45 | 4+3 | III | 3a | 0 | 0 | Heavy ion radiotherapy |
| 12 | PCa | 64 | 486 | 4+5 | IV | 4 | 1 | 1 | ADT |
| 13 | PCa | 74 | 60.8 | 5+5 | IV | 4 | 1 | 1 | ADT |
| 14 | PCa | 63 | 49.6 | 4+4 | IV | 3b | 1 | 0 | ADT |
| 15 | PCa | 69 | 1060 | 4+5 | IV | 4 | 1 | 1 | ADT |
| 16 | PCa | 67 | 34.9 | 4+4 | IV | 3b | 1 | 1 | ADT |
| 17 | PCa | 64 | 7.23 | 4+4 | IV | 3b | 1 | 0 | ADT |
| 18 | PCa | 79 | 3750 | 4+4 | IV | 3a | 0 | 1 | ADT |
| 19 | PCa | 78 | 1400 | 4+4 | IV | 3a | 0 | 1 | ADT |
| 20 | PCa | 77 | 2640 | 4+4 | IV | 4 | 1 | 1 | ADT |
| 21 | PCa | 69 | 554 | 4+4 | IV | 3b | 1 | 1 | ADT |
| 22 | PCa | 82 | 59.5 | 4+5 | III | 3b | 0 | 0 | ADT |
| 23 | PCa | 70 | 1030 | 4+4 | IV | 3b | 1 | 1 | ADT |
| 24 | PCa | 78 | 892 | 4+5 | IV | 4 | 1 | 1 | ADT |
| 25 | PCa | 86 | 24.2 | 4+4 | III | 3b | 0 | 0 | ADT |
| 26 | PCa | 67 | 64.6 | 4+5 | IV | 3b | 0 | 1 | ADT |
| 27 | PCa | 71 | 63.8 | 4+3 | III | 3a | 0 | 0 | Retropubic radical prostatectomy |
| 28 | PCa | 75 | 22.7 | 4+4 | III | 3b | 0 | 0 | ADT |
| 29 | PCa | 87 | 98.59 | 4+5 | IV | 4 | 1 | 0 | ADT |
| 30 | PCa | 78 | 693 | 4+4 | IV | 3b | 1 | 1 | ADT |
| 31 | PCa | 76 | 97.4 | 4+4 | IV | 3b | 1 | 1 | ADT |
| 32 | PCa | 64 | 1120 | 4+4 | IV | 4 | 1 | 1 | ADT |

| | | | | | | | | | |
|---|---|---|---|---|---|---|---|---|---|
| 33 | PCa | 78 | 266 | 4+4 | IV | 4 | 1 | 1 | ADT |
| 34 | PCa | 80 | 571 | 4+4 | IV | 4 | 1 | 1 | ADT |
| 35 | PCa | 65 | 277 | 4+5 | IV | 4 | 1 | 1 | ADT |
| 36 | PCa | 75 | 1000 | 4+5 | IV | 4 | 1 | 1 | ADT |
| 37 | PCa | 69 | 95.6 | 4+4 | IV | 4 | 1 | 1 | ADT |
| 38 | PCa | 66 | 36.08 | 4+5 | IV | 3a | 1 | 0 | ADT |
| 39 | PCa | 78 | 989 | 4+4 | IV | 4 | 1 | 1 | ADT |
| 40 | PCa | 73 | 478 | 4+3 | IV | 3b | 0 | 1 | ADT |
| 41 | PCa | 72 | 102 | 4+4 | III | 3a | 0 | 0 | ADT |
| 42 | PCa | 65 | 212 | 4+4 | IV | 4 | 1 | 1 | ADT |
| 43 | PCa | 81 | 11.4 | 4+4 | II | 2c | 0 | 0 | ADT |
| 44 | PCa | 73 | 467 | 4+4 | IV | 3b | 1 | 0 | ADT |
| 45 | PCa | 58 | 482 | 4+4 | IV | 3b | 1 | 0 | ADT |
| 46 | PCa | 73 | 76.4 | 4+4 | III | 3a | 0 | 0 | ADT |
| 47 | PCa | 79 | 63.2 | 4+5 | IV | 3b | 1 | 1 | ADT |
| 48 | PCa | 70 | 248 | 3+4 | IV | 3a | 1 | 0 | ADT |
| 49 | PCa | 81 | 1338 | 4+5 | IV | 4 | 0 | 1 | ADT |
| 50 | non-PCa | 69 | 12.2 | - | - | - | - | - | - |
| 51 | non-PCa | 66 | 11.9 | - | - | - | - | - | - |
| 52 | non-PCa | 85 | 10.1 | - | - | - | - | - | - |
| 53 | non-PCa | 55 | 11.2 | - | - | - | - | - | - |
| 54 | non-PCa | 67 | 22 | - | - | - | - | - | - |
| 55 | non-PCa | 66 | 7.33 | - | - | - | - | - | - |
| 56 | non-PCa | 69 | 7.92 | - | - | - | - | - | - |
| 57 | non-PCa | 68 | 7.24 | - | - | - | - | - | - |
| 58 | non-PCa | 53 | 4.33 | - | - | - | - | - | - |
| 59 | non-PCa | 62 | 5.14 | - | - | - | - | - | - |
| 60 | non-PCa | 63 | 10 | - | - | - | - | - | - |
| 61 | non-PCa | 62 | 5.11 | - | - | - | - | - | - |
| 62 | non-PCa | 74 | 8.3 | - | - | - | - | - | - |
| 63 | non-PCa | 65 | 4.3 | - | - | - | - | - | - |
| 64 | non-PCa | 72 | 5.43 | - | - | - | - | - | - |
| 65 | non-PCa | 66 | 5.35 | - | - | - | - | - | - |
| 66 | non-PCa | 67 | 5 | - | - | - | - | - | - |
| 67 | non-PCa | 66 | 19.5 | - | - | - | - | - | - |
| 68 | non-PCa | 63 | 12.9 | - | - | - | - | - | - |
| 69 | non-PCa | 53 | 6.69 | - | - | - | - | - | - |

| | | | | | | | | | |
|---|---|---|---|---|---|---|---|---|---|
| 70 | non-PCa | 63 | 5.3 | - | - | - | - | - | - |
| 71 | non-PCa | 66 | 7.73 | - | - | - | - | - | - |
| 72 | non-PCa | 57 | 7.3 | - | - | - | - | - | - |
| 73 | non-PCa | 54 | 5.44 | - | - | - | - | - | - |
| 74 | non-PCa | 67 | 5.93 | - | - | - | - | - | - |
| 75 | non-PCa | 67 | 8.09 | - | - | - | - | - | - |
| 76 | non-PCa | 60 | 14 | - | - | - | - | - | - |
| 77 | non-PCa | 69 | 5.99 | - | - | - | - | - | - |
| 78 | non-PCa | 62 | 5.62 | - | - | - | - | - | - |
| 79 | non-PCa | 52 | 4.41 | - | - | - | - | - | - |
| 80 | non-PCa | 63 | 9.19 | - | - | - | - | - | - |
| 81 | non-PCa | 60 | 5.6 | - | - | - | - | - | - |
| 82 | non-PCa | 56 | 8.44 | - | - | - | - | - | - |
| 83 | non-PCa | 61 | 8.6 | - | - | - | - | - | - |
| 84 | non-PCa | 62 | 35.5 | - | - | - | - | - | - |
| 85 | non-PCa | 73 | 6.02 | - | - | - | - | - | - |
| 86 | non-PCa | 57 | 5.19 | - | - | - | - | - | - |
| 87 | non-PCa | 64 | 4.37 | - | - | - | - | - | - |
| 88 | non-PCa | 60 | 5.68 | - | - | - | - | - | - |
| 89 | non-PCa | 63 | 11.4 | - | - | - | - | - | - |
| 90 | non-PCa | 65 | 13.2 | - | - | - | - | - | - |
