# Supplementary material for: The microRNA-23b/27b/24-1 cluster is a disease progression marker and tumor suppressor in prostate cancer
Source: Oncotarget. 2014 Jul 31;5(17):7748–59. doi: 10.18632/oncotarget.2294 (PMC4202158; doi:10.18632/oncotarget.2294)
Supplement: Supplementary file 2 [file oncotarget-05-7748-s002.docx]

Supplemental Table 2. Identification of target genes regulated by *miR-23b* in Pca

| Entrez gene ID | Symbol | Gene name | Location | Fold change | PC3 *miR-23b* transfectant |
| --- | --- | --- | --- | --- | --- |
| 51280 | GOLM1 | golgi membrane protein 1 | 9q21.33 | 1.82 | -1.38 |
| 957 | ENTPD5 | ectonucleoside triphosphate diphosphohydrolase 5 | 14q24.3 | 1.19 | -0.82 |
| 1767 | DNAH5 | dynein, axonemal, heavy chain 5 | 5p15.2 | 1.16 | -1.95 |
| 9185 | REPS2 | RALBP1 associated Eps domain containing 2 | Xp22.13 | 1.12 | -1.58 |
| 6695 | SPOCK1 | sparc/osteonectin, cwcv and kazal-like domains proteoglycan (testican) 1 | 5q31.2 | 1.11 | -1.09 |
| 6541 | SLC7A1 | solute carrier family 7 (cationic amino acid transporter, y+ system), member 1 | 13q12.3 | 0.99 | -0.57 |
| 100506658 | OCLN | occludin | 5q13.2 | 0.98 | -1.63 |
| 23389 | MED13L | mediator complex subunit 13-like | 12q24.21 | 0.89 | -0.69 |
| 23200 | ATP11B | ATPase, class VI, type 11B | 3q26.33 | 0.83 | -0.71 |
| 23576 | DDAH1 | dimethylarginine dimethylaminohydrolase 1 | 1p22.3 | 0.83 | -2.31 |
| 5589 | PRKCSH | protein kinase C substrate 80K-H | 19p13.2 | 0.82 | -0.75 |
| 129642 | MBOAT2 | membrane bound O-acyltransferase domain containing 2 | 2p25.1 | 0.76 | -1.45 |
| 1182 | CLCN3 | chloride channel 3 | 4q33 | 0.75 | -0.61 |
| 150864 | FAM117B | family with sequence similarity 117, member B | 2q33.2 | 0.73 | -0.65 |
| 23362 | PSD3 | pleckstrin and Sec7 domain containing 3 | 8p22 | 0.72 | -0.97 |
| 8829 | NRP1 | neuropilin 1 | 10p11.22 | 0.72 | -1.01 |
| 1387 | CREBBP | CREB binding protein | 16p13.3 | 0.72 | -0.52 |
| 124583 | CANT1 | calcium activated nucleotidase 1 | 17q25.3 | 0.71 | -0.57 |
| 8671 | SLC4A4 | solute carrier family 4, sodium bicarbonate cotransporter, member 4 | 4q13.3 | 0.70 | -2.37 |
| 7855 | FZD5 | frizzled family receptor 5 | 2q33.3 | 0.70 | -0.81 |
| 55839 | CENPN | centromere protein N | 16q23.2 | 0.68 | -1.23 |
| 7068 | THRB | thyroid hormone receptor, beta (erythroblastic leukemia viral (v-erb-a) oncogene homolog 2, avian) | 3p24.2 | 0.67 | -0.87 |
| 79017 | GGCT | gamma-glutamylcyclotransferase | 7p14.3 | 0.64 | -0.86 |
| 57674 | RNF213 | ring finger protein 213 | 17q25.3 | 0.64 | -0.57 |
| 140901 | STK35 | serine/threonine kinase 35 | 20p13 | 0.63 | -1.81 |
| 6418 | SET | SET nuclear oncogene | 9q34.11 | 0.63 | -0.56 |
| 54874 | FNBP1L | formin binding protein 1-like | 1p22.1 | 0.61 | -1.99 |
| 79048 | SECISBP2 | SECIS binding protein 2 | 9q22.2 | 0.60 | -1.67 |
| 288 | ANK3 | ankyrin 3, node of Ranvier (ankyrin G) | 10q21.2 | 0.60 | -1.77 |
| 5332 | PLCB4 | phospholipase C, beta 4 | 20p12.2 | 0.60 | -0.66 |
| 54894 | RNF43 | ring finger protein 43 | 17q22 | 0.60 | -2.04 |
| 27436 | EML4 | echinoderm microtubule associated protein like 4 | 2p21 | 0.59 | -1.49 |
| 57619 | SHROOM3 | shroom family member 3 | 4q21.1 | 0.59 | -0.72 |
| 8239 | USP9X | ubiquitin specific peptidase 9, X-linked | Xp11.4 | 0.59 | -0.78 |

Supplemental Table 3. Identification of target genes regulated by *miR-27b* in Pca

| Entrez gene ID | Symbol | Gene name | Location | Fold change | PC3 *miR-27b* transfectant |
| --- | --- | --- | --- | --- | --- |
| 51280 | GOLM1 | golgi membrane protein 1 | 9q21.33 | 1.82 | -2.00 |
| 10257 | ABCC4 | ATP-binding cassette, sub-family C (CFTR/MRP), member 4 | 13q32.1 | 1.24 | -0.60 |
| 28996 | HIPK2 | homeodomain interacting protein kinase 2 | 7q34 | 1.23 | -1.38 |
| 54848 | ARHGEF38 | Rho guanine nucleotide exchange factor (GEF) 38 | 4q24 | 1.18 | -1.84 |
| 1767 | DNAH5 | dynein, axonemal, heavy chain 5 | 5p15.2 | 1.16 | -1.74 |
| 100506658 | OCLN | occludin | 5q13.2 | 0.98 | -1.67 |
| 92292 | GLYATL1 | glycine-N-acyltransferase-like 1 | 11q12.1 | 0.96 | -0.56 |
| 81606 | LBH | limb bud and heart development homolog (mouse) | 2p23.1 | 0.90 | -0.74 |
| 2149 | F2R | coagulation factor II (thrombin) receptor | 5q13.3 | 0.86 | -0.56 |
| 19 | ABCA1 | ATP-binding cassette, sub-family A (ABC1), member 1 | 9q31.1 | 0.85 | -0.62 |
| 4628 | MYH10 | myosin, heavy chain 10, non-muscle | 17p13.1 | 0.84 | -0.97 |
| 22848 | AAK1 | AP2 associated kinase 1 | 2p13.3 | 0.84 | -1.01 |
| 51809 | GALNT7 | UDP-N-acetyl-alpha-D-galactosamine:polypeptide N-acetylgalactosaminyltransferase 7 (GalNAc-T7) | 4q34.1 | 0.83 | -1.20 |
| 23576 | DDAH1 | dimethylarginine dimethylaminohydrolase 1 | 1p22.3 | 0.83 | -2.59 |
| 490 | ATP2B1 | ATPase, Ca++ transporting, plasma membrane 1 | 12q21.33 | 0.82 | -1.82 |
| 1365 | CLDN3 | claudin 3 | 7q11.23 | 0.79 | -0.58 |
| 5140 | PDE3B | phosphodiesterase 3B, cGMP-inhibited | 11p15.2 | 0.76 | -0.61 |
| 65986 | ZBTB10 | zinc finger and BTB domain containing 10 | 8q21.13 | 0.75 | -0.99 |
| 64757 | MOSC1 | MOCO sulphurase C-terminal domain containing 1 | 1q41 | 0.75 | -1.70 |
| 2335 | FN1 | fibronectin 1 | 2q35 | 0.75 | -0.59 |
| 10103 | TSPAN1 | tetraspanin 1 | 1p34.1 | 0.75 | -0.61 |
| 1182 | CLCN3 | chloride channel 3 | 4q33 | 0.75 | -0.76 |
| 138050 | HGSNAT | heparan-alpha-glucosaminide N-acetyltransferase | 8p11.21 | 0.74 | -0.86 |
| 3329 | HSPD1 | heat shock 60kDa protein 1 (chaperonin) | 2q33.1 | 0.74 | -1.11 |
| 9693 | RAPGEF2 | Rap guanine nucleotide exchange factor (GEF) 2 | 4q32.1 | 0.73 | -0.66 |
| 23362 | PSD3 | pleckstrin and Sec7 domain containing 3 | 8p22 | 0.72 | -1.83 |
| 23657 | SLC7A11 | solute carrier family 7 (anionic amino acid transporter light chain, xc- system), member 11 | 4q28.3 | 0.72 | -1.88 |
| 114569 | MAL2 | mal, T-cell differentiation protein 2 (gene/pseudogene) | 8q24.12 | 0.72 | -1.33 |
| 9517 | SPTLC2 | serine palmitoyltransferase, long chain base subunit 2 | 14q24.3 | 0.71 | -0.86 |
| 389206 | BEND4 | BEN domain containing 4 | 4p13 | 0.70 | -0.65 |
| 8671 | SLC4A4 | solute carrier family 4, sodium bicarbonate cotransporter, member 4 | 4q13.3 | 0.70 | -1.24 |
| 58508 | MLL3 | myeloid/lymphoid or mixed-lineage leukemia 3 | 7q36.1 | 0.70 | -0.79 |
| 9969 | MED13 | mediator complex subunit 13 | 17q23.2 | 0.70 | -0.52 |
| 3837 | KPNB1 | karyopherin (importin) beta 1 | 17q21.32 | 0.69 | -1.16 |
| 319 | APOF | apolipoprotein F | 12q13.3 | 0.68 | -0.66 |
| 29927 | SEC61A1 | Sec61 alpha 1 subunit (S. cerevisiae) | 3q21.3 | 0.68 | -1.18 |
| 7068 | THRB | thyroid hormone receptor, beta (erythroblastic leukemia viral (v-erb-a) oncogene homolog 2, avian) | 3p24.2 | 0.67 | -0.84 |
| 57674 | RNF213 | ring finger protein 213 | 17q25.3 | 0.64 | -0.91 |
| 7157 | TP53 | tumor protein p53 | 17p13.1 | 0.63 | -0.73 |
| 2562 | GABRB3 | gamma-aminobutyric acid (GABA) A receptor, beta 3 | 15q12 | 0.63 | -0.96 |
| 2935 | GSPT1 | G1 to S phase transition 1 | 16p13.13 | 0.63 | -0.86 |
| 9781 | RNF144A | ring finger protein 144A | 2p25.1 | 0.63 | -0.69 |
| 93183 | PIGM | phosphatidylinositol glycan anchor biosynthesis, class M | 1q23.2 | 0.63 | -1.40 |
| 338645 | LUZP2 | leucine zipper protein 2 | 11p14.3 | 0.62 | -0.65 |
| 7058 | THBS2 | thrombospondin 2 | 6q27 | 0.62 | -0.65 |
| 80267 | EDEM3 | ER degradation enhancer, mannosidase alpha-like 3 | 1q25.3 | 0.62 | -1.06 |
| 54874 | FNBP1L | formin binding protein 1-like | 1p22.1 | 0.61 | -1.45 |
| 288 | ANK3 | ankyrin 3, node of Ranvier (ankyrin G) | 10q21.2 | 0.60 | -2.66 |
| 79822 | ARHGAP28 | Rho GTPase activating protein 28 | 18p11.31 | 0.60 | -1.84 |
| 58511 | DNASE2B | deoxyribonuclease II beta | 1p31.1 | 0.60 | -0.56 |
| 8322 | FZD4 | frizzled family receptor 4 | 11q14.2 | 0.59 | -0.64 |
| 8239 | USP9X | ubiquitin specific peptidase 9, X-linked | Xp11.4 | 0.59 | -1.87 |

Supplemental Table 4. Identification of target genes regulated by *miR-24* in Pca

| Entrez gene ID | Symbol | Gene name | Location | Fold change | PC3 *miR-24* transfectant |
| --- | --- | --- | --- | --- | --- |
| 31 | ACACA | acetyl-CoA carboxylase alpha | 17q12 | 1.27 | -1.29 |
| 9185 | REPS2 | RALBP1 associated Eps domain containing 2 | Xp22.13 | 1.12 | -1.40 |
| 56894 | AGPAT3 | 1-acylglycerol-3-phosphate O-acyltransferase 3 | 21q22.3 | 1.01 | -1.94 |
| 125058 | TBC1D16 | TBC1 domain family, member 16 | 17q25.3 | 1.01 | -1.54 |
| 6541 | SLC7A1 | solute carrier family 7 (cationic amino acid transporter, y+ system), member 1 | 13q12.3 | 0.99 | -0.72 |
| 56937 | PMEPA1 | prostate transmembrane protein, androgen induced 1 | 20q13.31 | 0.95 | -0.96 |
| 159195 | USP54 | ubiquitin specific peptidase 54 | 10q22.2 | 0.87 | -0.88 |
| 2149 | F2R | coagulation factor II (thrombin) receptor | 5q13.3 | 0.86 | -0.91 |
| 22848 | AAK1 | AP2 associated kinase 1 | 2p13.3 | 0.84 | -1.75 |
| 51313 | FAM198B | family with sequence similarity 198, member B | 4q32.1 | 0.83 | -2.13 |
| 23200 | ATP11B | ATPase, class VI, type 11B | 3q26.33 | 0.83 | -1.01 |
| 490 | ATP2B1 | ATPase, Ca++ transporting, plasma membrane 1 | 12q21.33 | 0.82 | -0.92 |
| 9761 | MLEC | malectin | 12q24.31 | 0.82 | -2.93 |
| 6158 | RPL28 | ribosomal protein L28 | 19q13.42 | 0.81 | -2.35 |
| 10525 | HYOU1 | hypoxia up-regulated 1 | 11q23.3 | 0.80 | -1.72 |
| 4299 | AFF1 | AF4/FMR2 family, member 1 | 4q22.1 | 0.78 | -0.95 |
| 4784 | NFIX | nuclear factor I/X (CCAAT-binding transcription factor) | 19p13.2 | 0.78 | -0.77 |
| 83988 | NCALD | neurocalcin delta | 8q22.3 | 0.77 | -1.57 |
| 10238 | DCAF7 | DDB1 and CUL4 associated factor 7 | 17q23.3 | 0.77 | -1.37 |
| 1182 | CLCN3 | chloride channel 3 | 4q33 | 0.75 | -2.50 |
| 138050 | HGSNAT | heparan-alpha-glucosaminide N-acetyltransferase | 8p11.21 | 0.74 | -0.56 |
| 10106 | CTDSP2 | CTD (carboxy-terminal domain, RNA polymerase II, polypeptide A) small phosphatase 2 | 12q14.1 | 0.73 | -1.59 |
| 9693 | RAPGEF2 | Rap guanine nucleotide exchange factor (GEF) 2 | 4q32.1 | 0.73 | -0.77 |
| 23362 | PSD3 | pleckstrin and Sec7 domain containing 3 | 8p22 | 0.72 | -1.15 |
| 8829 | NRP1 | neuropilin 1 | 10p11.22 | 0.72 | -1.09 |
| 1387 | CREBBP | CREB binding protein | 16p13.3 | 0.72 | -1.27 |
| 1729 | DIAPH1 | diaphanous homolog 1 (Drosophila) | 5q31.3 | 0.71 | -1.02 |
| 22992 | KDM2A | lysine (K)-specific demethylase 2A | 11q13.2 | 0.71 | -0.72 |
| 9517 | SPTLC2 | serine palmitoyltransferase, long chain base subunit 2 | 14q24.3 | 0.71 | -0.96 |
| 4642 | MYO1D | myosin ID | 17q11.2 | 0.71 | -1.67 |
| 57178 | ZMIZ1 | zinc finger, MIZ-type containing 1 | 10q22.3 | 0.70 | -0.61 |
| 9969 | MED13 | mediator complex subunit 13 | 17q23.2 | 0.70 | -0.91 |
| 3340 | NDST1 | N-deacetylase/N-sulfotransferase (heparan glucosaminyl) 1 | 5q33.1 | 0.69 | -1.19 |
| 55128 | TRIM68 | tripartite motif containing 68 | 11p15.4 | 0.68 | -0.53 |
| 9931 | HELZ | helicase with zinc finger | 17q24.2 | 0.68 | -1.09 |
| 55534 | MAML3 | mastermind-like 3 (Drosophila) | 4q31.1 | 0.67 | -1.05 |
| 2664 | GDI1 | GDP dissociation inhibitor 1 | Xq28 | 0.67 | -1.54 |
| 54020 | SLC37A1 | solute carrier family 37 (glycerol-3-phosphate transporter), member 1 | 21q22.3 | 0.67 | -1.07 |
| 5652 | PRSS8 | protease, serine, 8 | 16p11.2 | 0.66 | -1.48 |
| 2804 | GOLGB1 | golgin B1 | 3q13.33 | 0.65 | -1.77 |
| 3488 | IGFBP5 | insulin-like growth factor binding protein 5 | 2q35 | 0.64 | -1.20 |
| 9747 | FAM115A | family with sequence similarity 115, member A | 7q35 | 0.64 | -1.19 |
| 2562 | GABRB3 | gamma-aminobutyric acid (GABA) A receptor, beta 3 | 15q12 | 0.63 | -0.63 |
| 7311 | UBA52 | ubiquitin A-52 residue ribosomal protein fusion product 1 | 19p13.11 | 0.63 | -0.82 |
| 9781 | RNF144A | ring finger protein 144A | 2p25.1 | 0.63 | -1.58 |
| 10497 | UNC13B | unc-13 homolog B (C. elegans) | 9p13.3 | 0.63 | -2.06 |
| 10162 | LPCAT3 | lysophosphatidylcholine acyltransferase 3 | 12p13.31 | 0.61 | -2.43 |
| 55690 | PACS1 | phosphofurin acidic cluster sorting protein 1 | 11q13.2 | 0.61 | -1.13 |
| 79048 | SECISBP2 | SECIS binding protein 2 | 9q22.2 | 0.60 | -2.28 |
| 288 | ANK3 | ankyrin 3, node of Ranvier (ankyrin G) | 10q21.2 | 0.60 | -2.84 |
| 5332 | PLCB4 | phospholipase C, beta 4 | 20p12.2 | 0.60 | -1.06 |
| 27436 | EML4 | echinoderm microtubule associated protein like 4 | 2p21 | 0.59 | -0.65 |
| 57619 | SHROOM3 | shroom family member 3 | 4q21.1 | 0.59 | -1.58 |
| 2907 | GRINA | glutamate receptor, ionotropic, N-methyl D-aspartate-associated protein 1 (glutamate binding) | 8q24.3 | 0.59 | -1.35 |
| 8322 | FZD4 | frizzled family receptor 4 | 11q14.2 | 0.59 | -2.30 |
| 8239 | USP9X | ubiquitin specific peptidase 9, X-linked | Xp11.4 | 0.59 | -3.11 |
